# Supplementary material for: Obstructive sleep apnea and hypopnea syndrome in patients admitted in a tertiary hospital in Cameroon: Prevalence and associated factors
Source: PLoS One. 2020 Jan 16;15(1):e0227778. doi: 10.1371/journal.pone.0227778 (PMC6964861; doi:10.1371/journal.pone.0227778)
Supplement: Attachment — Submitted filename: Response to reviewer_R3_PO_OSAHS_YCH.docx [file pone.0227778.s004.docx]

To the Editors-in-Chief

Plos One

Submission of a revised manuscript

Manuscript ID: PONE-D-19-25768 entitled **" Obstructive sleep apnea and hypopnea syndrome in patients admitted in a tertiary hospital in Cameroon: prevalence and associated factors**"

Dear Editor,

We are grateful to the managing editor and reviewers for their time and comments on our manuscript (referenced above). We have addressed their comments and queries and would like to submit an updated version of our manuscript for publication in the Journal. Changes in the main document have been inserted with the use of red and blue color. In addition we are providing below a point-by-point response to each journal requirements.

**Additional Editor Comments**

Please amend your list of authors on the manuscript to ensure that each author is linked to an affiliation.

We note that you have included affiliation numbers 1-7, ¶ and & however only affiliations 1-7 have authors linked to them.  Please amend affiliation & and  ¶ to link an author to it or remove if added in error.

**Our answer:** This has been fixed.

We look forward to the outcome of our submission.

Sincerely,

Virginie Poka-Mayap, Dodo Balkissou Adamou, Massongo Massongo, Steve Voufouo Sonwa, Jacqueline Alime, Ben Patrick Michel Moutlen, Alfred Kongnyu Njamnshi, André Noseda And Eric Walter Pefura-Yone
